# Risk Factors for Hospitalizations Among Older Adults with Gastrointestinal Cancers

**DOI:** 10.1093/oncolo/oyab016

**Published:** 2022-01-28

**Authors:** Daneng Li, Can-Lan Sun, Rebecca Allen, Christiana J Crook, Abrahm Levi, Richard Ballena, Heidi D Klepin, Rawad Elias, Supriya G Mohile, William P Tew, Cynthia Owusu, Hyman B Muss, Stuart M Lichtman, Cary P Gross, Andrew E Chapman, Ajeet Gajra, Harvey J Cohen, Vani Katheria, Arti Hurria, William Dale

**Affiliations:** Department of Medical Oncology and Therapeutics Research, City of Hope, Duarte, CA, USA; Patient and Family Resource Center, City of Hope, Duarte, CA, USA; Department of Medical Oncology and Therapeutics Research, City of Hope, Duarte, CA, USA; Department of Medical Oncology and Therapeutics Research, City of Hope, Duarte, CA, USA; Department of Medical Oncology and Therapeutics Research, City of Hope, Duarte, CA, USA; Department of Medical Oncology and Therapeutics Research, City of Hope, Duarte, CA, USA; Department of Internal Medicine, Section on Hematology and Oncology, Wake Forest Baptist Comprehensive Cancer Center, Winston-Salem, NC, USA; Department of Medical Oncology, Hartford Healthcare Cancer Institute, Hartford, CT, USA; Department of Medicine, Hematology/Oncology, University of Rochester Medical Center, Rochester, NY, USA; Department of Gynecologic Medical Oncology, Memorial Sloan Kettering Cancer Center, New York, NY, USA; Department of Medicine, School of Medicine, Case Western University School of Medicine, Cleveland, OH, USA; Geriatric Oncology Program, Division of Oncology, Lineberger Comprehensive Cancer Center, University of North Carolina at Chapel Hill, Chapel Hill, NC, USA; Department of Gynecologic Medical Oncology, Memorial Sloan Kettering Cancer Center, New York, NY, USA; Cancer Outcomes Public Policy and Effectiveness Research (COPPER) Center, Yale School of Medicine, New Haven, CT, USA; Department of Medical Oncology, Sidney Kimmel Cancer Center/Jefferson Health, Philadelphia, PA, USA; Cardinal Health, Dublin, OH, USA; SUNY Upstate Medical University, Syracuse, NY, USA; Center for the Study of Aging and Human Development, Duke University School of Medicine, Durham, NC, USA; Center for Cancer and Aging, City of Hope, Duarte, CA, USA; Department of Medical Oncology and Therapeutics Research, City of Hope, Duarte, CA, USA; Department of Population Sciences, City of Hope, Duarte, CA, USA; Department of Supportive Care, City of Hope, Duarte, CA, USA

**Keywords:** geriatric assessment, gastrointestinal, cancer, hospitalization

## Abstract

**Background:**

Older adults (≥65 years) with gastrointestinal (GI) cancers who receive chemotherapy are at increased risk of hospitalization caused by treatment-related toxicity. Geriatric assessment (GA) has been previously shown to predict risk of toxicity in older adults undergoing chemotherapy. However, studies incorporating the GA specifically in older adults with GI cancers have been limited. This study sought to identify GA-based risk factors for chemotherapy toxicity–related hospitalization among older adults with GI cancers.

**Patients and Methods:**

We performed a secondary post hoc subgroup analysis of two prospective studies used to develop and validate a GA-based chemotherapy toxicity score. The incidence of unplanned hospitalizations during the course of chemotherapy treatment was determined.

**Results:**

This analysis included 199 patients aged ≥65 years with a diagnosis of GI cancer (85 colorectal, 51 gastric/esophageal, and 63 pancreatic/hepatobiliary). Sixty-five (32.7%) patients had ≥1 hospitalization. Univariate analysis identified sex (female), cardiac comorbidity, stage IV disease, low serum albumin, cancer type (gastric/esophageal), hearing deficits, and polypharmacy as risk factors for hospitalization. Multivariable analyses found that patients who had cardiac comorbidity (OR 2.48, 95% CI 1.13-5.42) were significantly more likely to be hospitalized.

**Conclusion:**

Cardiac comorbidity may be a risk factor for hospitalization in older adults with GI cancers receiving chemotherapy. Further studies with larger sample sizes are warranted to examine the relationship between GA measures and hospitalization in this vulnerable population.

## Teaser

A major health care cost for older adults with GI cancers is hospitalization. This article identifies geriatric assessment risk factors for chemotherapy toxicity-related hospitalization among older adults with gastrointestinal cancers.

**Implications for Practice**

There are a lack of data whether geriatric assessment (GA) variables are associated with hospitalization during chemotherapy among older adults (≥65 years) with gastrointestinal cancers. This study examined GA variables to identify hospitalization risk factors within this population. Univariate analysis identified sex (female), cardiac comorbidity, stage IV disease, low serum albumin, cancer type (gastric/esophageal), hearing deficits, and polypharmacy as significant risk factors for hospitalization, of which cardiac comorbidity remained a significant risk factor on multivariable analysis. Additional studies with larger sample sizes are needed to better assess the role of the GA in this vulnerable population.

## Introduction

Cancer is a disease of aging; however, older adults are underrepresented in cancer clinical studies.^1-4^ Of the cancer types that affect older adults, gastrointestinal (GI) cancers are particularly common.^5-7^ A major health care cost for older adults with GI cancers is hospitalization.^5^ In prior studies, potential risk factors for hospitalization among older adults with GI cancers include receipt of radiation therapy,^8^ advanced disease stage,^5^ and comorbidities such as diabetes or chronic obstructive pulmonary disease.^9^ However, data regarding additional patient-specific measures that can impact hospitalization risk in this population are lacking.

Geriatric assessment (GA) is a comprehensive tool, validated in older adults receiving chemotherapy, that evaluates physical function, comorbidity, nutritional status, polypharmacy, social support, cognition, and psychological status.^10,11^ Geriatric assessment has demonstrated the ability to predict chemotherapy toxicity^4^ and risk of hospitalization^12^ among older adults with solid tumors. In particular, having a diagnosis of GI cancer was identified as a risk for increased toxicity in older adults receiving chemotherapy.^4,13^ However, studies specifically using the GA to identify hospitalization risk factors among older adults with GI cancers are limited. Identification of risk factors for hospitalization in this patient population will enable the development of GA-driven interventional trials to potentially allow physicians to better address these risk factors in a preventive and proactive manner in hopes of avoiding hospitalizations in this vulnerable population.

To address this gap in knowledge, we performed a pooled subgroup analysis of older adults with GI cancers who received chemotherapy and enrolled previously on prospective cohort studies aimed to develop^4^ and validate^13^ a predictive model of severe chemotherapy toxicity in older adults. The primary objective of this analysis was to identify risk factors associated with chemotherapy toxicity–related unplanned hospitalization in older adults with GI cancers. We hypothesized that impairment in GA measures would be associated with increased risk of chemotherapy toxicity–related unplanned hospitalization.

## Materials and Methods

### Study Design and Participants

**Figure 1.**
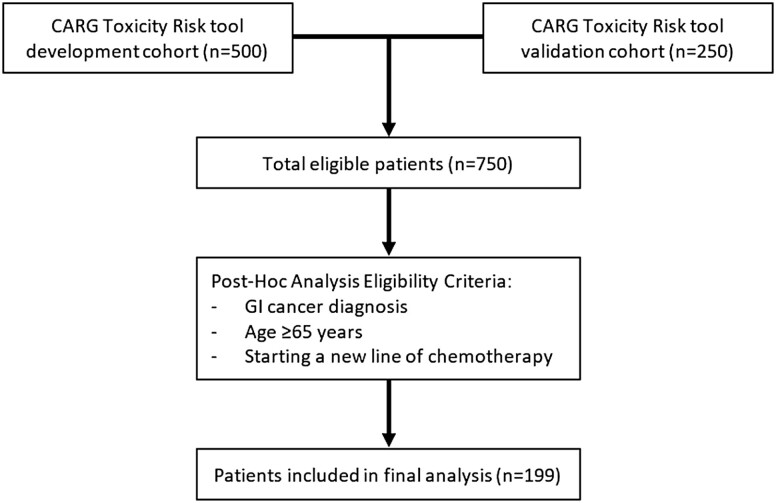
CONSORT flow diagram for inclusion of patients in analysis.

Secondary analysis was performed using data obtained from patients who participated in IRB-approved multi-site prospective cohort studies to develop^4^ (*n* = 500) and validate^13^ (*n* = 250) a GA-based chemotherapy toxicity score for older adults with cancer. Eligibility requirements for the current study were as follows: age ≥65 years, GI tumor diagnosis, starting a new chemotherapy regimen, and fluency in English (not all survey measures had been validated in languages other than English) (Figure 1). The City of Hope IRB provided approval for conducting this analysis; the parent studies were approved by all sites’ institutional review boards. All participants provided informed consent.

### Measures

The primary outcome measure of this secondary analysis was incident unplanned hospitalization(s) during chemotherapy treatment, excluding any planned or scheduled admissions. Patients were followed from initiation of chemotherapy until the last day of the last cycle or 12 months after initiation of chemotherapy, whichever occurred first. Data on hospitalization were abstracted from the medical records at each site and reviewed by two physicians. This process included review of notes, discharge summaries, phone notes, and scanned media to capture hospitalizations that happened within or outside of the institution’s electronic medical records system. The treating physician was queried to resolve any uncertainty. Reasons for hospitalization were recorded and adjudicated by two-physician review. If more than one reason for hospitalization was listed, the highest-grade toxicity resulting in hospitalization was reported.

### Data Collection

Clinical data, abstracted from the medical record, included routinely collected laboratory values (albumin, white cell count, blood urea nitrogen, creatinine, and hepatic function tests), cancer type, stage, treatment (single agent vs. multi-agent; standard dose vs. dose reduction at first cycle), use of growth factors, and receipt of prior chemotherapy. Creatinine clearance was calculated using the Jelliffe formula with ideal body weight. Participants completed a GA before starting chemotherapy. The GA includes a health care provider–administered assessment and a self-administered patient questionnaire.^4,13^ The health care provider–administered questionnaire included the following assessments: (1) Karnofsky performance status (KPS)^14^; (2) Timed Up and Go (a performance-based measure of physical function; time assessed in seconds for those who could complete the test or recorded as unable to perform)^15^; (3) Blessed Orientation-Memory-Concentration (BOMC) test (score ≥11 indicating cognitive impairment)^16^; and (4) recording of height and weight (current and 6 months prior) to evaluate nutritional status, including calculation of body mass index.

The self-administered patient questionnaire included the following surveys: self-reported measures of physical function and activities (Activities of Daily Living [ADL] subscale of Medical Outcomes Study [MOS] Physical Health survey and the Instrumental Activities of Daily Living [IADL] subscale of the Older Americans Resources and Services [OARS] survey),^17,18^ a patient-rated KPS,^14^ self-reported falls in the past 6 months, self-reported comorbid conditions and a rating of the degree to which each causes interference in activities (Physical Health Section subscale of the OARS survey),^17^ number and type of medications, assessment of psychological state (symptoms of anxiety and depression using the Mental Health Inventory-17),^19^ social activity, and social support (MOS Social Activity and Social Support surveys).^18,20^

### Statistical Analysis

We calculated descriptive statistics summarizing patient demographics, clinical characteristics, and GA domains. Univariate logistic regression models were used to obtain odds ratios (ORs), corresponding 95% confidence intervals (CIs), and *P*-values for each variable associated with hospitalization. These variables included demographic/disease characteristics, GA measures of function, comorbidity, cognition, nutrition, and psychosocial status, and laboratory values including albumin, hemoglobin, creatinine clearance, white cell count, and liver function tests. Given the fixed sample size (*n* = 199 patients) and our expectation that 30% of patients would be hospitalized, the current study would have 80% power at a two-sided α value of 0.05 to detect minimally detected odds ratios of 2.27 to 2.72 for risk factors with a prevalence of 20% to 50%.

Variables with a univariate *P*-value <.05 were included in the multivariable logistic regression. We examined bi-variable correlations among all univariate variables with *P*-values <.1 and they were not highly correlated (data not shown). Clinically meaningful variables, such as age and treatment with poly-chemotherapy, were included in the multivariable model regardless of *P*-value. All statistical tests were two-sided and *P*-values <.05 were considered statistically significant. Data were analyzed using SAS 9.4 analytic software (SAS Institute, Cary, NC).

## Results

### Patient Characteristics and Hospitalizations

A total of 199 patients between 65 and 94 years old (median age 73 years) with GI cancers were included in this secondary analysis. Patient characteristics are described in Table 1. One hundred eleven patients (55.8%) were female, 160 (80.4%) were White, and 117 (58.8%) reported completion of college or post-college education. In terms of primary tumor diagnosis, 85 (42.7%) were diagnosed with colorectal cancer, 51 (25.6%) with gastric/esophageal cancer, and 63 (31.7%) with pancreatic/hepatobiliary cancer. Among them, 83 (42.7%) were diagnosed with stages I-III disease and 116 (58.3%) were diagnosed with stage IV disease. The majority of patients received first-line chemotherapy (75.4%), poly-chemotherapy (66.8%), and at standard dose (57.8%); granulocyte colony-stimulating factor was not used for most patients (77.9%) (Table 1). Eighty-nine patients (44.7%) received 5-fluorouracil (5-FU)-based chemotherapy, 50 (25.1%) received gemcitabine-based chemotherapy, and 60 (30.2%) received another type of chemotherapy.

**Table 1.** Baseline patient characteristics in relation to unplanned hospitalization during chemotherapy.

	Unplanned hospitalization during chemotherapy			
	No	Yes	Total	*P*-value
Number of patients	134	65	199	
Age (years)				.19
Mean (SD)	73.3 (6.43)	74.7 (6.96)	73.7 (6.63)	
Median (range)	72 (65-94)	75 (65-89)	73 (65-94)	
Sex, *n* (column%)				.02
Female	67 (50.0%)	44 (67.7%)	111 (55.8%)	
Male	67 (50.0%)	21 (32.3%)	88 (44.2%)	
Race/ethnicity, *n* (column%)				.17
White	104 (77.6%)	56 (86.2%)	160 (80.4%)	
Black	13 (9.7%)	4 (6.2%)	17 (8.5%)	
Asian	9 (6.7%)	5 (7.7%)	14 (7.0%)	
Other	8 (6.0%)	0 (0.0%)	8 (4.0%)	
Education, *n* (column%)				.59
High school or less	52 (38.8%)	30 (46.2%)	82 (41.2%)	
College graduate	54 (40.3%)	24 (36.9%)	78 (39.2%)	
Advanced degree	28 (20.9%)	11 (16.9%)	39 (19.6%)	
Marital status, *n* (column%)				.08
Married	81 (60.4%)	48 (73.8%)	129 (64.8%)	
Widowed	24 (17.9%)	11 (16.9%)	35 (17.6%)	
Single/separated/divorced	29 (21.6%)	6 (9.2%)	35 (17.6%)	
Household composition, *n* (column%)				.66
Alone	24 (18.2%)	10 (15.6%)	34 (17.1%)	
With someone	108 (81.8%)	54 (84.4%)	162 (81.4%)	
Missing	2	1	3	
Employment, *n* (column%)				.47
Employed	25 (18.7%)	12 (18.8%)	37 (18.6%)	
Retired, homemaker, unemployed	102 (76.1%)	51 (79.7%)	153 (76.9%)	
Disabled/medical leave	7 (5.2%)	1 (1.6%)	8 (4.0%)	
Missing	0	1	1	
Body mass index (kg/m^2^)				.81
Median (range)	25.2 (15.2-44.0)	24.8 (19.9-36.9)	25.0 (15.2-44.0)	
Missing	0	1	1	
Cancer type, *n* (column%)				.08
Colorectal	64 (47.8%)	21 (32.3%)	85 (42.7%)	
Gastric/esophageal	29 (21.6%)	22 (33.8%)	51 (25.6%)	
Pancreatic/hepatobiliary	41 (30.6%)	22 (33.8%)	63 (31.7%)	
Cancer stage, *n* (column%)				.08
I/II	21 (15.7%)	5 (7.7%)	26 (13.1%)	
III	42 (31.3%)	15 (23.1%)	57 (28.6%)	
IV	71 (53.0%)	45 (69.2%)	116 (58.3%)	
Line of chemotherapy, *n* (column%)				.73
First line	102 (76.1%)	48 (73.8%)	150 (75.4%)	
>First line	32 (23.9%)	17 (26.2%)	49 (24.6%)	
Number of chemotherapy agents, *n* (column%)				.62
Single	46 (34.3%)	20 (30.8%)	66 (33.2%)	
Poly	88 (65.7%)	45 (69.2%)	133 (66.8%)	
Standard dose, *n* (column%)				.86
No	56 (41.8%)	28 (43.1%)	84 (42.2%)	
Yes	78 (58.2%)	37 (56.9%)	115 (57.8%)	
Use of granulocyte colony-stimulating factor, *n* (column%)				
No	107 (79.9%)	48 (73.8%)	155 (77.9%)	.34
Yes	27 (20.1%)	17 (26.2%)	44 (22.1%)	

SD is a standard abbreviation. *P*-values were obtained from *t*-test for continuous variables and chi-square test for categorical variables.

Among the 199 patients, 65 (32.7%) were hospitalized during the course of chemotherapy. Of the hospitalized patients, 55 patients were hospitalized once; 9 patients were hospitalized twice; 1 patient was hospitalized 3 times. Length of hospitalization could be calculated for 60 patients; 5 patients had unknown discharge dates, making it not possible to accurately calculate length of stay. For those evaluable for a first hospitalization, the median duration of stay was 4.5 days with a range of 1 to 66 days. Among the 10 patients hospitalized for a second time, the median length of stay was 2.5 days with a range of 1 to 7 days. The patient hospitalized for a third time had a stay of one day. Reasons for incident hospitalization are presented in Table 2. The most common reasons for hospitalization were infection (40.0%), GI disorders (18.5%), cardiovascular problems (10.8%), and dehydration (10.8%).

**Table 2.** Primary reason for incident hospitalization during chemotherapy. *N* = 65 patients.

Reason for hospitalization	*n*
Infection with or without neutropenia, *n* (%)	26 (40.0%)
Infection with normal ANC	14
Infection without normal ANC	6
Fever/infection	6
Gastrointestinal disorders, n (%)	12 (18.5%)
Bowel perforation	1
Constipation	2
Dehydration/syncope/diarrhea	1
Diarrhea	2
Gastrointestinal toxicity	1
Nausea	4
Vomiting	1
Cardiovascular, *n* (%)	7 (10.8%)
Cardiac ischemia/infarction	1
Cardiac troponin elevated	1
Cerebrovascular ischemia	1
Supraventricular arrythmia	2
Hypotension	1
Cardiopulmonary arrest/atrial fibrillation	1
Dehydration, *n* (%)	7 (10.8%)
Dehydration	6
Dehydration/infection with normal ANC	1
Thrombosis, *n* (%)	4 (6.2%)
Thrombosis/embolism	2
Thrombosis/embolism, infection with normal ANC	1
Pulmonary embolism, CNS cerebrovascular ischemia	1
Respiratory, *n* (%)	2 (3.1%)
Bronchospasm	1
Pulmonary/upper respiratory, other	1
Bleeding, *n* (%)	4 (6.2%)
Gastrointestinal bleeding	1
Hemorrhage	3
Neuropathy, *n* (%)	1 (1.5%)
Other (hypokalemia), *n* (%)	1 (1.5%)
Unknown, *n* (%)	1 (1.5%)

Abbreviations: ANC, absolute neutrophil count; CNS, central nervous system.

### Factors Associated with Hospitalization

We did not observe significant associations between ADL, IADL, KPS, Timed Up and Go, number of falls in the past 6 months, psychological state, social activity, social support, nutrition, BOMC score, and hospitalization (Table 3). However, on univariate analysis, seven other factors were found to be significantly associated with increased odds of hospitalization: female sex, diagnosis of stage IV disease, diagnosis of gastric/esophageal cancer, polypharmacy (≥5 daily medications), decreased hearing, patient-reported cardiac comorbidity (history of heart disease), and low serum albumin (<3.5 g/dL) (Table 4). Although age and poly-chemotherapy were not significant in univariate analysis, they were included in the multivariable model as predefined clinically meaningful variables. In multivariable analysis, patients with self-reported cardiac comorbidity (OR 2.48, 95% CI 1.13-5.42, *P*-value = 0.02) were still significantly more likely to be hospitalized, while hearing deficits, polypharmacy, cancer type (gastric/esophageal), and female sex became nonsignificant after adjustment for other variables (Table 4). Among the 65 hospitalized patients, 25 patients reported pre-existing cardiac comorbidity. Of the seven patients hospitalized for cardiac toxicity (one cardiac ischemia/infarction, one cardiac troponin elevated, one cerebrovascular ischemia, two supraventricular arrythmia, one hypotension, and one cardiopulmonary arrest/atrial fibrillation), four had self-reported pre-existing cardiac comorbidity.

**Table 3.** Associations between geriatric assessment variables and unplanned hospitalization during chemotherapy.

	Unplanned hospitalization during chemotherapy		Univariate	
	No (*N* = 134)	Yes (*N* = 65)	OR (95% CI)	*P*-value
ADL score (mean (SD))	69.8 (26.20)	64.8 (29.37)	0.99 (0.98-1.00)	0.23
IADL score (mean (SD))	12.7 (2.11)	12.5 (2.30)	0.95 (0.83-1.09)	0.49
Self-reported KPS (mean (SD))	84.7 (13.94)	82.7 (15.64)	0.99 (0.97-1.01)	0.37
MD-reported KPS (mean (SD))	84.7 (12.89)	83.0 (13.88)	0.99 (0.97-1.01)	0.40
Timed Up and Go (mean (SD))	12.3 (4.84)	13.5 (7.64)	1.03 (0.98-1.09)	0.26
Number of falls in past 6 months (mean (SD))	0.5 (3.06)	0.4 (1.10)	0.97 (0.85-1.12)	0.72
HADS anxiety (mean (SD))	4.6 (3.39)	4.8 (3.92)	1.02 (0.94-1.12)	0.64
HADS depression (mean (SD))	3.9 (3.33)	3.7 (2.81)	0.98 (0.89-1.08)	0.70
Social activity (mean (SD))	54.2 (24.47)	56.3 (22.63)	1.00 (0.99-1.02)	0.55
Social support (mean (SD))	86.4 (19.66)	88.5 (16.88)	1.01 (0.99-1.02)	0.46
Tangible (mean (SD))	86.3 (21.88)	88.0 (17.93)	1.00 (0.99-1.02)	0.37
Emotional (mean (SD))	86.5 (20.32)	88.8 (18.20)	1.00 (0.99-1.02)	0.59
Body mass index (mean (SD))	25.6 (5.00)	25.4 (3.69)	0.99 (0.93-1.06)	0.80
BOMC score ≥11, *n* (%)	11 (8.2%)	6 (9.2%)	1.14 (0.40-3.22)	0.81

ADL, activities of daily living; IADL, instrumental activities of daily living; KPS, Karnofsky performance status; HADS, Hospital Anxiety and Depression Scale; BOMC, Blessed Orientation-Memory-Concentration; SD, standard deviation; OR, odds ratio; CI, confidence interval. *P*-values were obtained from *t*-test for continuous variables and chi-square test for categorical variables.

**Table 4.** Risk factors associated with unplanned hospitalization during chemotherapy.

	Unplanned hospitalization during chemotherapy		Univariate		Multivariable	
	No (%) (*N* = 134)	Yes (%) (*N* = 65)	OR (95% CI)	*P*-value	OR (95% CI)	*P*-value
Age (years) (mean (SD))	73.3 (6.43)	74.7 (6.96)	1.03 (0.98-1.08)	0.16	1.03 (0.98-1.09)	0.23
Sex, *n* (row%)						
Male	67 (76.1%)	21 (23.9%)	1.0		1.0	
Female	67 (60.4%)	44 (39.6%)	2.10 (1.13-3.90)	0.02	1.80 (0.89-3.65)	0.10
Cancer type, *n* (row%)						
Colorectal	64 (75.3%)	21 (24.7%)	1.0		1.0	
Gastric/esophageal	29 (56.9%)	22 (43.1%)	1.92 (1.03-3.56)	0.03	2.15 (0.90-5.16)	0.09
Pancreatic/hepatobiliary	41 (63.1%)	22 (34.9%)	1.63 (0.80-3.34)	0.18	1.78 (0.74-4.29)	0.20
Cancer stage, *n* (row%)						
I/II/III	63 (75.9%)	20 (24.1%)	1.0		1.0	
IV	71 (61.2%)	45 (38.8%)	2.00 (1.07-3.74)	0.03	1.57 (0.78-3.18)	0.21
Number of chemotherapy agents, *n* (row%)						
Single	46 (65.7%)	20 (30.3%)	1.0		1.0	
Poly	88 (66.2%)	45 (33.8%)	1.18 (0.62-2.22)	0.62	1.75 (0.75-4.10)	0.20
Hearing problem, *n* (row%)						
No	105 (70.9%)	43 (29.1%)	1.0		1.0	
Yes	28 (56.0%)	22 (44.0%)	1.92 (1.00-3.72)	0.05	2.05 (0.95-4.43)	0.07
Missing	1	0				
History of heart disease, *n* (row%)						
No	114 (74.0%)	40 (26.0%)	1.0		1.0	
Yes	20 (44.4%)	25 (55.6%)	3.56 (1.79-7.10)	<0.001	2.48 (1.13-5.42)	0.02
Daily medications, *n* (row%)						
0-4	90 (75.0%)	30 (25.0%)	1.0		1.0	
5+	42 (56.8%)	32 (43.2%)	2.29 (1.23-4.24)	0.008	1.88 (0.94-3.75)	0.07
Missing	2	3				
Serum albumin, *n* (row%)						
Normal ([3.5 g/dL, 5.5 g/dL])	95 (72.0%)	37 (28.0%)	1.0		1.0	
Low (<3.5 g/dL)	39 (58.2%)	28 (41.8%)	1.84 (1.00-3.41)	0.05	1.64 (0.81-3.32)	0.17

Multivariable ORs were obtained from models with all variables adjusted in the model.

## Discussion

In this study, we observed that patient-reported cardiac comorbidity was a risk factor for unplanned hospitalization during chemotherapy among older adults with GI cancer. While several other GA variables were identified as risk factors for hospitalization on univariate analysis, these variables did not remain significant on multivariable analysis.

Pre-existing cardiac comorbidity was reported by 45 out of 199 patients (22.6%). Of these 45 patients, 25 (55.6%) were hospitalized during their chemotherapy treatment. It is possible that pre-existing cardiac comorbidity places a cancer patient at greater risk for a variety of chemotherapy toxicity–related events that could result in hospitalization. For example, Kadlec et al. identified heart failure, atrial fibrillation, and hypertension as risk factors for thromboembolic events in patients with lung cancer^21^ and Khorana et al. identified arterial thromboembolism as a risk factor for venous thromboembolic events in a retrospective cohort study of hospitalized cancer patients.^22^ In addition, previous studies have shown that 5-FU-based chemotherapy can contribute to increased risk of cardiotoxicity in older adults, particularly those with cardiac comorbidities.^23-26^ Interestingly, in our small hypothesis-generating study, four of the seven patients hospitalized for cardiovascular toxicity had received 5-FU-based chemotherapy. Of these four patients, three were hospitalized during cycle one and one was hospitalized during cycle two. The increased potential for cardiovascular and thromboembolic events may be a contributing factor to the increased risk of hospitalization among this population. Therefore, investigating the cardiac history of older adults with GI cancers, particularly those who are planning to receive 5-FU-based chemotherapy, is warranted to better identify risk factors for hospitalization among this potentially vulnerable population.

Polypharmacy, hearing deficits, cancer type (gastric/esophageal), and female sex were significant risk factors for hospitalization on univariate analysis; however, our study did not have enough power to determine their significance on multivariable analysis. Klepin et al. recently identified disease type (GI cancers), low serum albumin, reduced creatinine clearance, polypharmacy, increased number of comorbidities, and needing help with instrumental activities of daily living as hospitalization risk factors in their study of older adults with solid tumors who received chemotherapy.^12^ Given that Klepin et al. identified GI cancers as a risk factor for hospitalization in older adults receiving chemotherapy, it is possible that the symptomatic nature of GI cancers could be driving this hospitalization risk. Another possible reason for the differences between the results of our study and those of Klepin et al. may be that our post hoc subgroup analysis was not sufficiently powered to detect the significance of some of the GA variables as potential risk factors for hospitalization. For example, consistent with Klepin et al., low serum albumin and polypharmacy were significant univariate variables in our study. Hearing deficits and low serum albumin are also risk factors for chemotherapy toxicity in the original Cancer and Aging Research Group chemotherapy toxicity score developed from the parent study population used in this analysis.^4,13^ Given that our current analysis is hypothesis-generating, these risk factors remain potentially relevant in predicting hospitalization risk. Therefore, performing larger prospective studies specifically within the older adult GI cancer population with sufficient power are needed in order to better identify the role of GA variables as additional risk factors for hospitalization. Alternatively, modifications of the GA may be needed in order to more accurately identify risk factors for hospitalization among this population. This would help to improve the design of future GA-driven interventional trials that could potentially prevent chemotherapy toxicity-related hospitalizations among older adults with GI cancers.

There are several strengths of our study. First, our study population was focused on older adults with GI cancers, whose hospitalization risk factors have not been extensively studied before. Second, our study used a validated and standardized outpatient GA,^4,10,13^ allowing us to capture additional vulnerabilities specific to older adults undergoing chemotherapy. Third, our study identified risk factors outside of age alone as potential predictors of hospitalization in this vulnerable patient population of older adults with GI cancers. Limitations of our post hoc hypothesis-generating study include its relatively small sample size (199 patients, 65 of whom had recorded hospitalizations), which limited the power to detect potential significant findings. As a consequence of our small sample size and the secondary nature of our analysis, residual confounders may not have been accounted for in our final analyses. The small sample size of our study also prevented us from performing meaningful subgroup analyses for specific reasons of hospitalization. Additionally, the self-reported nature of the GA implies that patients’ answers could have potential response bias. While we were able to capture whether or not a patient reported pre-existing cardiac comorbidity, the parent studies did not capture the type or severity of these comorbidities, which could have impacted the results of our analyses. However, self-report has been used to capture patient comorbidity in many studies,^12,27-31^ demonstrating its clinical utility. Ultimately, given that patient-reported cardiac comorbidity was identified as a risk factor for hospitalization among older adults with GI cancers in the current analysis, future studies assessing the role of GA for identifying hospitalization risk factors in this population will include more detailed assessments of patients’ cardiac comorbidity at the time of study enrollment.

## Conclusions

Patient-reported cardiac comorbidity is a risk factor for hospitalization among older adults with GI cancers undergoing chemotherapy. Sex (female), hearing deficits, polypharmacy, low serum albumin, disease type (gastric/esophageal), and disease stage (IV) may be additional risk factors for hospitalization, warranting further investigation. Large prospective studies focused specifically on identification of GA measures as risk factors for hospitalization in this vulnerable population of older adults with GI cancers are needed to help inform the appropriate design of future GA-driven interventional studies within this population.

## Data Availability

The data underlying this article will be shared on reasonable request to the corresponding author.
